# Validation of the Chinese Version of the HPV Stigma Scale: A Multidimensional Analysis in Women With HPV

**DOI:** 10.1002/brb3.71044

**Published:** 2025-11-11

**Authors:** Zhenwei Dai, Shu Jing, Haiyan Hu, Tianjie Yang, Yanzhu Wang, Dongxue Chen, Feifei Gao, Xue Han, Zhaorui Liu, Xiaoyou Su, Youlin Qiao

**Affiliations:** ^1^ Peking University Sixth Hospital Peking University Institute of Mental Health NHC Key Laboratory of Mental Health (Peking University) National Clinical Research Center for Mental Disorders (Peking University Sixth Hospital) Beijing China; ^2^ School of Population Medicine and Public Health Chinese Academy of Medical Sciences and Peking Union Medical College Beijing China; ^3^ Shenzhen Maternity and Child Healthcare Hospital Southern Medical University Guangzhou Guangdong China

**Keywords:** adaptation, HPV, scale, stigma, validation

## Abstract

**Background:**

Human papillomavirus (HPV) infection is a global public health issue, and HPV‐related stigma can affect cervical cancer prevention. But no validated tools exist to assess HPV stigma in Chinese adult women infected with HPV. This study aimed to adapt and validate the HPVsStigma scale (HPV‐SS) in the Chinese context.

**Methods:**

A cross‐sectional study was conducted from December 2024 to February 2025 among 501 HPV‐infected women in Shenzhen, China. The HPV‐SS was adapted from a 12‐item HIV stigma scale. Demographic characteristics, HPV‐related variables, and data on mental health were collected. Factor analyses (FA) were used to assess the scale's factorial structure, reliability, and validity. The bi‐factor model was used to determine the score‐reporting method of the scale. Item response theory (IRT) was employed to assess the relationship between participants’ stigma levels and scale scores. Latent profile analysis (LPA) was conducted to classify the participants with different HPV stigma characteristics and determine the optimal cut‐off value for HPV‐SS.

**Results:**

FA showed that the 3‐factor model (personalized stigma, public‐disclosure concerns, and negative self‐image) had the best fit among the nested models, with good reliability and validity. The bi‐factor model analysis indicated that the total scale score was more meaningful than dimension scores. IRT analysis confirmed that higher HPV‐SS scores represented higher stigma levels. LPA identified a 2‐class model as optimal, and the optimal cut‐off value of the scale for high HPV stigma was 35.

**Conclusion:**

This study validated the 12‐item HPV‐SS for Chinese women infected with HPV, with good reliability and validity. The scale can be used to evaluate HPV stigma levels, facilitating targeted interventions to improve cervical cancer prevention and the psychological well‐being of affected women.

## Graphical abstract

This study employs factor analysis, bi‐factor modeling, item response theory, and latent profile analysis, revealing good reliability and validity of HPV stigma scale among women infected with HPV in China.

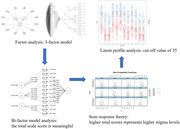

## 1 Introduction

HPV infection has posed a persistent public health challenge worldwide, and approximately 80% of sexually active women and men will experience at least one HPV infection during their lifetime (Milano et al. 2023). The World Health Organization (WHO) reported that over 95% of cervical cancer cases are attributable to HPV infection (WHO 2024). In 2022, cervical cancer was ranked as the fourth leading cause of cancer morbidity and mortality among women worldwide, and 23% of new cases and 16% of deaths from cervical cancer were recorded in China (Wu et al. 2025). A nationwide study with medical institutions’ data from 2017 to 2023 showed an overall HPV prevalence of 17.70% among Chinese women, with 13.12% being high‐risk HPV infections (Han et al. 2025). Although ensuring the accessibility, affordability, and quality of cervical cancer prevention services such as screening, treatment, and vaccination is undoubtedly essential, the stigmatizing attitudes within the community characterizing HPV‐positive women as promiscuous or unfaithful may intensify the perceived stigma experienced by HPV‐infected women, which may result in a diminished perception of risk and a reduced possibility of accepting screening and treatment (Li et al. 2025, Ginjupalli et al. 2022).

Stigma refers to the process of assigning negative labels to certain groups, which leads to stereotyping, separation, status loss, and discrimination (Gyamfi 2024). A study in Western Kenya implied that for women infected with HPV, misinformation and insufficient knowledge about HPV can exacerbate their internalized stigma (Ginjupalli et al. 2022). Studies have emphasized that HPV‐related stigma has significantly impeded cervical cancer prevention initiatives. A study conducted in Peru indicated that HPV‐related stigma is widespread in women infected with HPV, which can result in delayed cancer screening, treatment avoidance, and even premature mortality due to the progression of cervical cancer (Morse et al. 2023). A review in the United States also indicated that women infected with HPV are vulnerable to high levels of stigma caused by social stereotyping and prejudice, fear of social judgment, and self‐blame. Such intense stigma can disrupt personal relationships, distort self‐image, and foster a sense of alienation, which can lead to reluctance in HPV vaccination uptake, cervical cancer screening, and seeking timely interventions on the prevention of the progression from HPV infection to cervical cancer (Peterson et al. 2021).

To comprehensively understand the HPV‐related stigma in women infected with HPV, validated measurement tools are indispensable for accurately assessing their levels of HPV‐related stigma. A study in the United States developed an HPV‐related stigma scale among sexually experienced girls based on a scale measuring HIV‐related stigma in adults, and this scale has also been applied in Canadian university students (Kahn et al. 2008, Jones et al. 2016). A study in London, UK, adapted an 8‐item HPV stigma scale for female university students based on a previous sexually transmitted diseases (STD) stigma scale (Waller et al. 2007). An investigation in Hong Kong, China developed a 7‐item scale for healthcare workers to evaluate their stigmatizing beliefs towards individuals with high‐risk HPV infection (Kwan et al. 2012). Despite these previous efforts, research focusing on the HPV stigma experienced by adult women (aged 18 years and older) infected with HPV remains scarce, and in the context of China, no validated tools have been developed to assess HPV stigma in women infected with HPV. To fill this gap, the presen t study is aimed at adapting and validating an HPV‐SS within the Chinese cultural context, to deepen the understanding of the characteristics of HPV stigma and improve the evaluation of HPV stigma among Chinese adult women infected with HPV.

## 2 Methods

### 2.1 Sampling and Participants

This cross‐sectional study was conducted and reported in accordance with the Strengthening the Reporting of Observational Studies in Epidemiology (STROBE) guidelines. This study was conducted among women infected with HPV in Shenzhen, Guangdong Province, China, from December 2024 to February 2025. The study population of HPV‐infected women was recruited from the Cervical Department of a maternity and child healthcare hospital in Shenzhen by convenience sampling. The eligibility criteria were: (1) women testing positive for HPV according to standard laboratory tests; (2) aged 18 years and over; (3) having the ability to complete questionnaires independently; and (4) providing voluntary informed consent to participate in this study. Women with significant cognitive impairment were excluded from the study. The questionnaire was rigorously developed and validated through a multi‐stage process. An expert panel comprising epidemiologists, clinical psychologists, physicians, and nursing specialists designed the self‐administered online instrument based on an extensive literature review and a series of structured focus group discussions. Content validity was established through iterative revisions and expert evaluation, ensuring relevance, clarity, and comprehensiveness of all items in measuring the target constructs within the study population. The online survey platform “Sojump” was utilized to administer the questionnaire, and all questions were set as mandatory fields (wjx.cn). In total, 501 questionnaires were collected. After carefully examining the response times and logical consistencies of the questionnaires, none were excluded from further analysis, with an effective recovery rate of 100.0%. This study was approved by the Ethics Committee of the Chinese Academy of Medical Science (ID: CAMS&PUMC‐IEC‐2024‐001) on January 12th, 2024.

### 2.2 Measures

#### 2.2.1 Demographic Characteristics

Demographic characteristics included age, ethnicity, permanent residence in the past six months, religion, degree, marriage status, annual household income, alcohol consumption, number of children, and sexual partners. Additionally, variables associated with the HPV infection were also collected, including whether the participants had received the HPV vaccine or undergone regular cervical cancer screening before the diagnosis, disease staging, and discomfort state during the treatment.

#### 2.2.2 HPV‐SS

The HPV‐SS was modified from the 12‐item HIV stigma scale that was developed by Reinius et al. in 2017 that has been translated and validated in the Chinese cultural context (Li et al. 2015, Reinius et al. 2017, Berger et al. 2001). The HPV‐SS has 12 items and each item was Likert‐scaled from 1 to 5. Higher total scores indicate higher levels of HPV stigma. The Cronbach's α of this scale was 0.953 in this study.

#### 2.2.3 Depression and Anxiety Symptoms

The depression and anxiety scales were used to evaluate the concurrent validity of HPV‐SS (Ho et al. 2015). The depression was measured by the 9‐item Patient Health Questionnaire (PHQ‐9). This questionnaire has been validated among various Chinese populations (Yao et al. 2021, Hou et al. 2021, Kroenke et al. 2001). The items of the PHQ‐9 are scored on a 4‐point Likert scale with a range of 0 to 3. Higher total scores indicated higher levels of depression symptoms, and a total score of equal to or above 10 can indicate depression symptoms. The Cronbach's α of the instrument in this study was 0.934. The anxiety was measured by the Generalized Anxiety Disorder Questionnaire (GAD‐7). This scale consists of seven items that are rated on a 4‐point Likert scale from 0–3. Higher total scores indicated higher levels of anxiety symptoms, and a total score of equal to or above 10 can indicate anxiety symptoms. This instrument is reliable and valid among the Chinese population (Miao et al. 2021, Gong et al. 2021). The Cronbach's α of the instrument in this study was 0.956.

### 2.3 Adaptation of the HPV‐SS

The adaptation of the HPV‐SS adhered to a systematic and multi‐step process. Given the conceptual and theoretical overlap between HIV stigma and HPV stigma, the 12‐item HIV‐SS was selected as the foundation for adaptation. Both HIV and HPV are sexually transmitted infections that share significant commonalities in their potential to elicit stigma, including associations with sexual behavior, fears of contagion, and social judgment (Li et al. 2015, Reinius et al. 2017, Berger et al. 2001). We first replaced the “HIV” with “HPV” in the 12‐item HIV‐SS. These items were then critically evaluated by a panel of experts in HPV, oncology, and psychometrics to ensure consistency with the specific characteristics and experiences associated with women infected with HPV and enhance the scale's content validity. Finally, a 12‐item HPV‐SS was initially adapted with four dimensions as per the dimensions of HIV‐SS: personalized stigma, disclosure concerns, concerns about public attitudes, and negative self‐image. Considering the potential similarities between the two dimensions of disclosure concerns and concerns about public attitudes, we merged these two dimensions into one dimension named public‐disclosure concerns in the subsequent analyses to determine the optimal dimension constitution of the scale.

### 2.4 Statistical Analysis

Descriptive analyses were conducted to describe the demographic characteristics of the study population. FA were used to analyze the factorial structure of HPV‐SS. In FA, the dataset was randomly split into two subsets using the random number generator in Excel, with one used for exploratory factor analysis (EFA) and the other for confirmatory factor analysis (CFA). In the EFA, the maximum likelihood (ML) extraction method was employed in combination with varimax rotation to initially identify the underlying factorial structure and loadings (Revelle 2024).

For the CFA, the ML estimation method was applied, and construct validity was assessed using model fit indices, including the χ^2^ test, comparative fit index (CFI), Tucker‐Lewis index (TLI), root mean square error of approximation (RMSEA), and standardized root mean square residual (SRMR). The criteria for a good model fit are: non‐significant χ^2^ test (*p* > 0.05), CFI > 0.9, TLI > 0.9, RMSEA < 0.08, and SRMR < 0.08. Additionally, three nested models were built for comparison to determine the optimal factorial structure: a 1‐factor model (one dimension), a 3‐factor model (personalized stigma, public‐disclosure concerns, and negative self‐image), and a 4‐factor model (personalized stigma, disclosure concerns, concerns about public attitudes, and negative self‐image). The parsimony normed fit index (PNFI) and parsimony goodness of fit index (PGFI) were additionally used for comparison between nested models. The criteria for a good nested model are: PNFI > 0.5 and PGFI > 0.5, with higher values indicating a better model. Convergent validity was evaluated using Composite Reliability (CR) and average variance extracted, with CR > 0.7 and AVE > 0.5 indicating good convergent validity. Discriminant validity was assessed via the heterotrait‐monotrait ratio of correlations (HTMT), with all HTMT ratio values < 0.9 indicating good discriminant validity (Rosseel 2012, Jorgensen et al. 2022). Concurrent validity was evaluated by the correlation between the optimal factors of HPV‐SS, the total scores of the PHQ‐9, and GAD‐7, where significant correlations indicated good concurrent validity. Multi‐group CFA with full dataset stratified by disease stages was used to evaluate the factorial equivalence of the scale across the four disease stages. Multiple‐indicator multiple‐cause (MIMIC) model analysis was further conducted to evaluate the factorial invariance of HPV‐SS across multiple demographic characteristics.

The best‐fitting CFA model was subsequently used to fit the bi‐factor model with the full dataset, where unidimensionality was examined using the percentage of uncontaminated correlations (PUC) or explained common variance (ECV), with PUC > 0.7 or ECV > 0.7 indicating unidimensionality. CR and the homogeneity coefficient (ωH) were used to evaluate the rationality of reporting total scale scores or subscale scores. Specifically, total scale and subscale scores were considered meaningful if total scale ωH > 0.5, subscale CR > 0.7, and subscale ωH/CR > 0.7; only total scale scores were meaningful if total scale ωH > 0.5 but subscale CR < 0.7 or ωH/CR <0.7; only subscale scores were considered meaningful if total scale ωH < 0.5 and subscale CR > 0.7; and neither score was considered meaningful in other cases (Honglei and Zhonglin 2017).

IRT with graded response model (GRM) was then employed to assess the relationship between participants’ stigma levels and scale total scores and item scores, with the relationships visualized through item characteristic curves (ICC), expected total score plot, and expected item score plot (Chalmers 2012).

Finally, LPA was conducted with 12 items as observed variables to further classify the participants with different HPV stigma characteristics. Models with 1 to 5 latent classes were specified, and the optimal model was selected based on a comprehensive comparison of fit indices, including Akaike information criterion (AIC), Bayesian information criterion (BIC), consistent Akaike information criterion (CAIC), sample‐size‐adjusted BIC (SABIC), and entropy. Lower values of AIC, BIC, CAIC, and SABIC indicate better model fit. An entropy > 0.9 indicates high classification accuracy. Model selection will also consider the practical implications and parsimony of the latent class structure (Rosenberg et al. 2018). Based on the LPA results, receiver operating characteristic (ROC) curve analysis and Youden's index were used to determine the scale's optimal cut‐off values (Thiele and Hirschfeld 2021). All statistical analyses were performed using R 4.4.2 and Mplus 8.3 (R Core Team 1998–2017, 2024).

## 3 Results

### 3.1 Demographic Characteristics

A total of 501 females with HPV infection were included in this study, with an average age of 39.7 ± 8.6. Demographic characteristics showed that 94.2% were of Han ethnicity, and 95.6% lived in urban areas. Regarding socioeconomic status, 29.7% held associate degrees, 54.1% reported an annual household income of CNY 100,000–300,000, 59.5% reported no alcohol consumption, 83.4% were married, 40.5% had one child, and 91.8% maintained monogamous sexual relationships. Healthcare‐related data revealed that 70.7% had received HPV vaccination and 86.4% underwent regular cervical cancer screenings before diagnosis, 34.9% were diagnosed with high‐grade cervical lesions, and 83.4% reported no treatment‐related discomfort. The results are shown in Table 1.

**TABLE 1:** Demographic characteristics.

Variables	Mean (SD) or *n* (%)
Age	39.7 ± 8.6
Ethnicity	—
Han	472 (94.2)
Other	29 (5.8)
Permanent residence in the past 6 months	
Urban area	479 (95.6)
Rural area	22 (4.4)
Religion	
No	474 (94.6)
Yes	27 (5.4)
Degree	—
Junior high school degree or below	52 (10.4)
Senior high school or secondary vocational school degree	118 (23.6)
Associate degree	149 (29.7)
Bachelor's degree	139 (27.7)
Master's or doctor's degree	43 (8.6)
Marriage Status	
Single/unmarried	55 (11.0)
Married	418 (83.4)
Divorced	28 (5.6)
Annual household income (ten thousand yuan)	
≤ 5	10 (2.0)
5–10	71 (14.2)
10–30	271 (54.1)
> 30	149 (29.7)
Alcohol	
No	298 (59.5)
Yes	203 (40.5)
Number of children	—
0	74 (14.8)
1	203 (40.5)
2	191 (38.1)
≥ 3	33 (6.6)
Number of sexual partners	—
0	6 (1.2)
1	460 (91.8)
≥ 2	35 (7.0)
HPV vaccination	
No	147 (29.3)
Yes	354 (70.7)
Regular cervical cancer screening before diagnosis	
No	68 (13.6)
Yes	433 (86.4)
Disease staging	
HPV infection	132 (26.4)
Low‐grade squamous intraepithelial lesions	167 (33.3)
High‐grade squamous intraepithelial lesions	175 (34.9)
Cervical cancer	27 (5.4)
Discomfort during the treatment	—
No	418 (83.4)
Yes	83 (16.6)

### 3.2 EFA

The Kaiser‐Meyer‐Olkin (KMO) measure of sampling adequacy of HPV‐SS was 0.9, and Bartlett's test of sphericity showed a *p*‐value of less than 0.001, supporting the appropriateness of conducting FA. In EFA, only 1 factor was extracted, with factor loadings ranging from 0.7 to 0.9, indicating the high reliability of all items. However, the EFA model had a poor model fit: RMSEA = 0.200, TLI = 0.775, and considering the data‐driven nature of EFA and the potential factor structure of HPV‐SS mentioned before, several nested models that included the 1‐factor model will be compared in the subsequent CFA. The details of the scale items and factor loadings in the EFA are given in Supplementary Table 1.

### 3.3 CFA

#### 3.3.1 Reliability and Validity Analyses

The 1‐factor model (one dimension), 3‐factor model (personalized stigma, public‐disclosure concerns, and negative self‐image), and 4‐factor model (personalized stigma, disclosure concerns, concerns about public attitudes, and negative self‐image) were respectively built for comparison. The 1‐factor model showed poor model fit (χ^2^ test *p* < 0.001, CFI = 0.821, TLI = 0.782, RMSEA = 0.185, SRMR = 0.056) due to high residual correlations. While the 3‐factor and 4‐factor models had acceptable model fit (All χ^2^ test *p* < 0.001, CFI = 0.941 v.s. 0.950, TLI = 0.924 v.s. 0.931, RMSEA = 0.109 v.s. 0.104, and SRMR = 0.064 v.s. 0.062). However, the PNFI (0.714 v.s. 0.679) and PGFI (0.547 v.s. 0.517) of the 4‐factor model were both lower than those of the 3‐factor model, indicating the 3‐factor model may have optimal structural validity. The model fit of the three models is summarized in Table 2.

**TABLE 2:** Model fit of the three nested models.

	1‐factor model	3‐factor model	4‐factor model	Criteria
χ^2^	517	203	177	—
df	54	51	48	—
P	< 0.001	< 0.001	< 0.001	> 0.05
CFI	0.821	0.941	0.950	> 0.9
TLI	0.782	0.924	0.931	> 0.9
RMSEA	0.185	0.109	0.104	< 0.08
SRMR	0.056	0.064	0.062	< 0.08
PNFI	0.659	0.714	0.679	> 0.5
PGFI	0.550	0.547	0.517	> 0.5

The factor loadings for the 1‐factor CFA model ranged from 0.7 to 0.9, for the 3‐factor CFA model also from 0.6 to 1.0, and for the 4‐factor CFA model from 0.6 to 1.0. All factor loadings were statistically significant, indicating strong internal consistency and good reliability of the items across all three models. The CR values were all above 0.7, and the AVE values were all above 0.5 in the three models, indicating good convergent validity of the three models. The details are shown in Table 3 and Supplementary Tables 2–3.

**TABLE 3:** Parameter estimates, reliability, and convergent validity of the 3‐factor model.

Factor	Indicator	Estimate	SE	β	z	*p*	CR	AVE
F1	HSS1	1.0	—	0.6	—	—	0.9	0.7
	HSS2	1.7	0.1	1.0	12.4	< 0.001	—	—
—	HSS3	1.5	0.1	0.9	12.1	< 0.001	—	—
F2	HSS4	1.0	—	0.7	—	—	0.9	0.7
—	HSS5	1.4	0.1	0.9	12.2	< 0.001	—	—
—	HSS6	1.1	0.1	0.8	10.9	< 0.001	—	—
—	HSS7	1.5	0.1	0.9	12.5	< 0.001	—	—
—	HSS8	1.4	0.1	0.9	12.5	< 0.001	—	—
—	HSS9	1.2	0.1	0.8	11.0	< 0.001	—	—
F3	HSS10	1.0	—	0.7	—	—	0.9	0.7
—	HSS11	1.3	0.1	0.9	12.5	< 0.001	—	—
	HSS12	1.2	0.1	0.9	12.3	< 0.001		

Abbreviations: F1, Personalized stigma; F2, Public‐disclosure concerns; F3, Negative self‐image; HSS1‐HSS12, 12 items of the HPV stigma scale; CR, composite reliability; AVE, average variance extracted.

**FIGURE 1:**
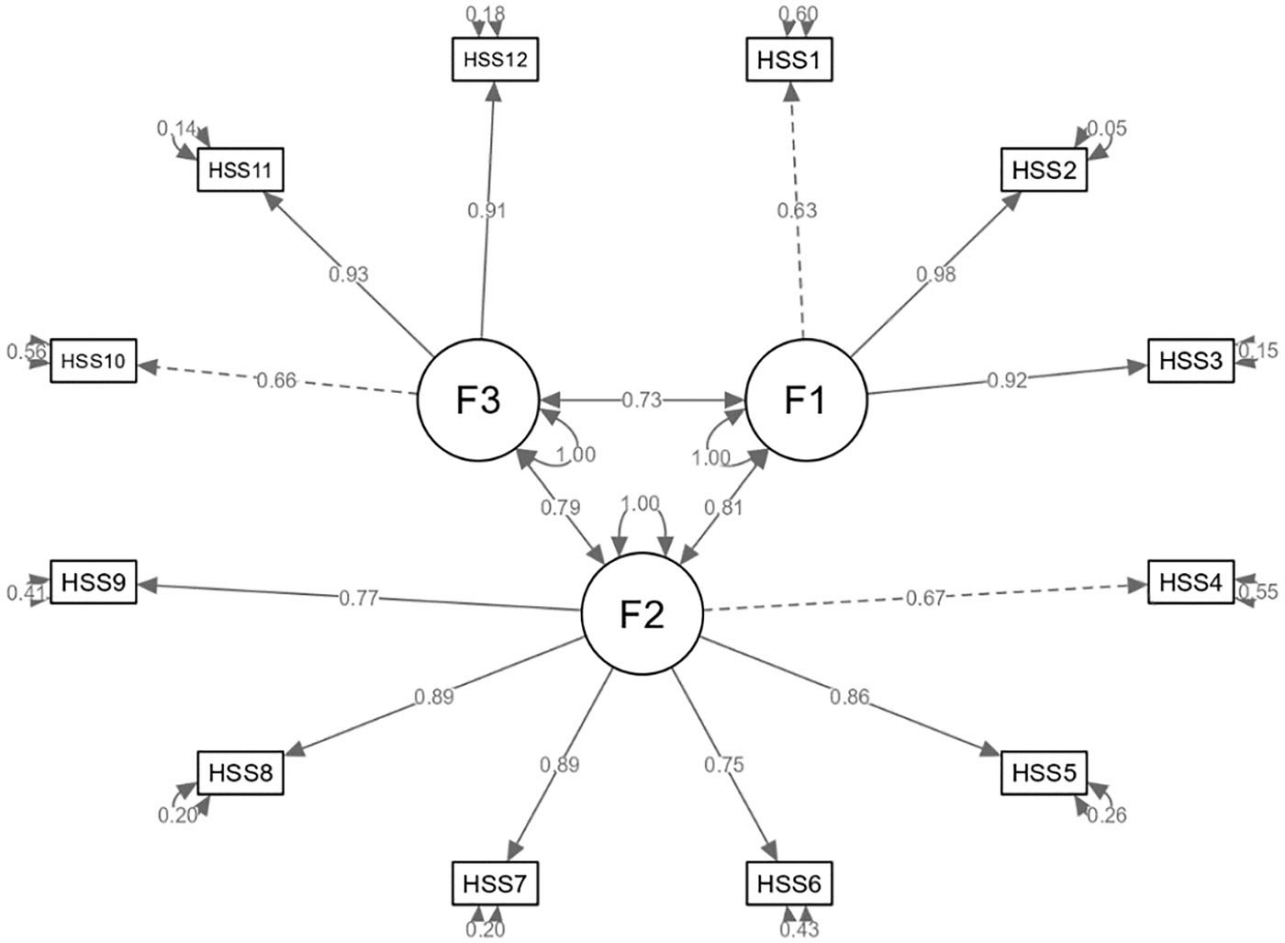
Three‐factor CFA model of the HPV stigma scale.

For the discriminant validity, the HTMT ratio value between disclosure concerns and concerns about public attitudes was 0.909 in the 4‐factor model, indicating poor discriminant validity of this model due to the high correlation between the two dimensions. While the HTMT ratio values in the 3‐factor models were all below 0.9, indicating acceptable discriminant validity of the 3‐factor model. See Table 4 and Supplementary Table 4. Based on the comparison of structural, convergent, and discriminant validity in the 3 nested models, we determined the 3‐factor model to be the optimal model. The correlations between the 3 factors and depression and anxiety ranged from 0.6 to 0.7, and were all statistically significant, indicating good concurrent validity of the 3‐factor model, see Table 5. The factor structure of the 3‐factor model is given in Figure 1.

**TABLE 4:** Heterotrait‐monotrait ratio of correlations in the 3‐factor model.

	F1	F2	F3
F1	1	—	—
F2	0.885	1	—
F3	0.821	0.837	1

Abbreviations: F1, Personalized stigma; F2, Public‐disclosure concerns; F3, Negative self‐image.

**TABLE 5:** Concurrent validity of the 3‐factor model.

	F1	F2	F3	Depression	Anxiety
F1	1	—	—	—	—
F2	0.8***	1***	—	—	—
F3	0.7***	0.7***	1	—	—
Depression	0.6***	0.6***	0.7***	1	—
Anxiety	0.6***	0.6***	0.7***	0.9***	1

Abbreviations: F1, Personalized stigma; F2, Disclosure concerns; F3, Concerns about public attitudes; F4, Negative self‐image; ***: *p* < 0.001.

#### 3.3.2 Multi‐Group Analyses

To assess potential heterogeneity in scale performance across disease stages (HPV infection, low‐grade squamous intraepithelial lesions, high‐grade squamous intraepithelial lesion, and cervical cancer), multi‐group CFA stratified by disease stage was conducted to evaluate factorial invariance across the four stages. Six nested models with progressively constrained parameters were tested: a baseline model without constraints, a metric invariance model, a scalar invariance model, a measurement residual variance invariance model, a factor variance and covariance invariance model, and a latent mean invariance model. Although the χ^2^ tests were statistically significant for all constrained models (all *p* < 0.001), suggesting a lack of strict statistical invariance, changes in the TLI were all below the recommended threshold of 0.05 (Cheung and Rensvold 2002). This indicates that the scale can be considered functionally equivalent across disease stages, supporting its invariant psychometric properties among women with varying severity of HPV‐related conditions. The results are shown in Table 6.

**TABLE 6:** Multi‐group analyses on disease stages.

Model	Δχ^2^	Δdf	*p*	ΔTLI
Baseline	—	—	—	—
Metric invariance	76.3	27	< 0.001	−0.006
Scalar invariance	141.3	36	< 0.001	0.002
Measurement residual variance invariance	373.8	36	< 0.001	0.041
Factor variance and covariance invariance	107.0	18	< 0.001	0.004
Latent mean invariance	163.0	9	< 0.001	0.019

Abbreviations: df, degree of freedom; TLI, Tucker‐Lewis Index.

#### 3.3.3 MIMIC Model Analyses

**FIGURE 2:**
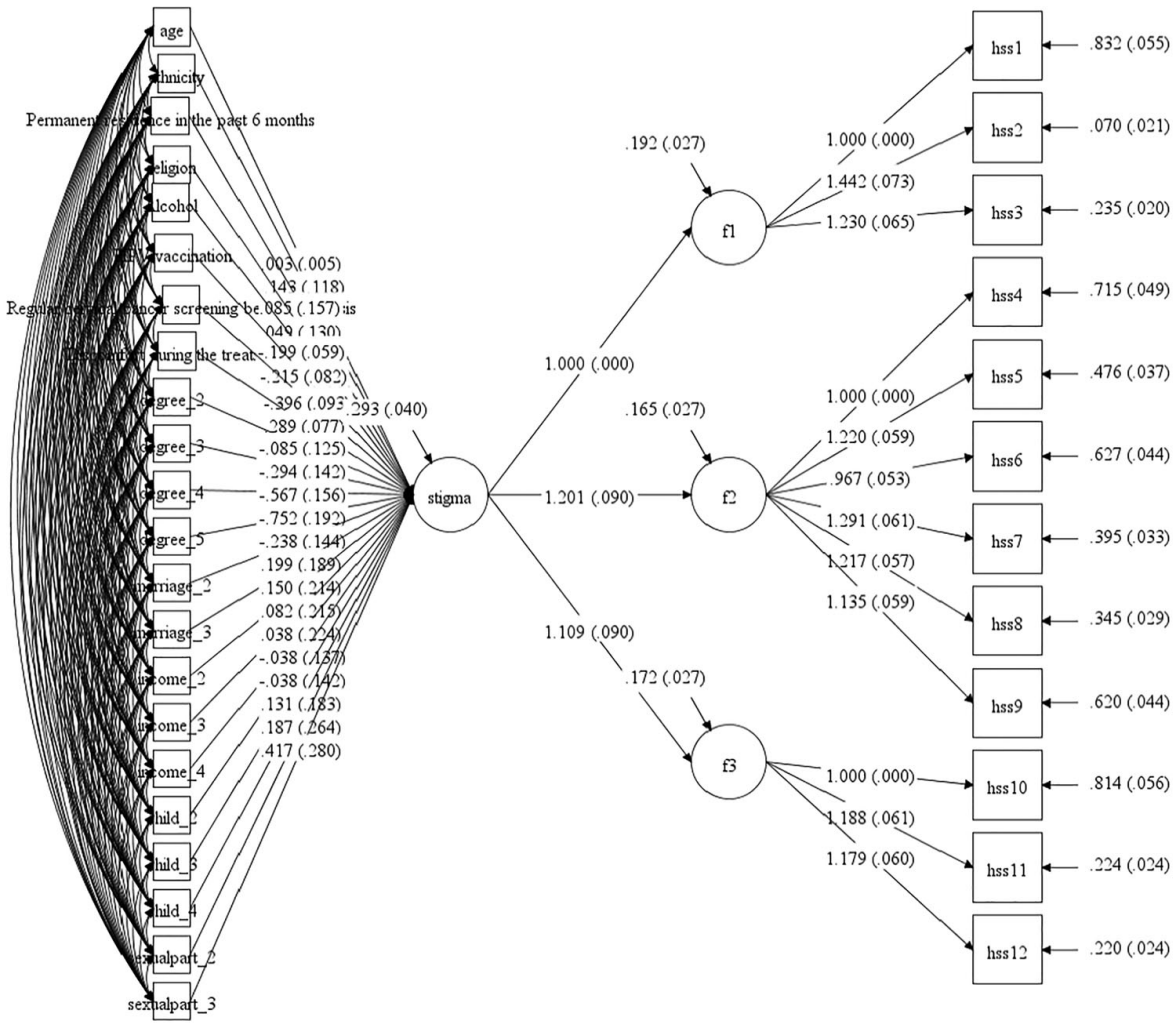
MIMIC model on the factorial invariance of HPV Stigma Scale across demographic characteristics.

A MIMIC model was established to assess the factorial invariance of HPV‐SS across various demographic characteristics of the participants. The model showed good model fit: CFI = 0.913, TLI = 0.902, RMSEA = 0.059, and SRMR = 0.047. The analysis revealed that the psychometric properties of the HPV‐SS varied significantly depending on participants’ educational level, alcohol use, HPV vaccination status, history of cervical cancer screening prior to diagnosis, and experience of discomfort during treatment. Detailed results of the MIMIC model are presented in Figure 2 and Supplementary Table 5. However, to evaluate practical rather than strict statistical invariance, multi‐group CFAs were subsequently conducted for each demographic variable identified as significant in the MIMIC model. The results indicated that all ΔTLI values were below the 0.05 threshold, supporting the practical factorial invariance of the HPV‐SS across these demographic characteristics. Detailed results from the multi‐group CFAs are provided in Supplementary Tables 6–10.

#### 3.3.4 Sensitivity Analysis

A sensitivity analysis was performed to assess the potential influence of the small yet clinically severe cervical cancer subgroup (*n* = 27) on the robustness of the overall model. After excluding these cases, the 3‐factor CFA model was re‐estimated. While the RMSEA value (0.125) exceeded the conventional threshold of 0.08, it remained within a reasonable range for complex psychological constructs, and all other fit indices indicated good model fit: CFI = 0.927, TLI = 0.906, and SRMR = 0.066. These results support the robustness of the factor structure and suggest that the overall model is not influenced by the characteristics of this distinct subgroup.

### 3.4 Bi‐factor Model Analysis

The 3‐factor CFA model was used to fit the bi‐factor model. The PUC and ECV were both above 0.7, suggesting unidimensionality of the scale. The ωH of the total scale was 0.9, and the CR values of the three dimensions were all above 0.7. While ratios of ωH to CR for each dimension were below 0.7, indicating that reporting aggregate scores for the entire scale is more meaningful than reporting scores for individual dimensions. These findings are presented in Table 7.

**TABLE 7:** Factor loadings and relative statistics of the bi‐factor model.

Indicator	Bi‐factor model					
—	HPV stigma	F1	F2	F3	ECV	PUC
HSS1	0.7	0.1			0.8	0.7
HSS2	0.8	0.8	—	—		
HSS3	0.7	0.4	—	—		
HSS4	0.8	—	0.3	—		
HSS5	0.9	—	0.2	—		
HSS6	0.8	—	0.3	—		
HSS7	0.9	—	−0.1	—		
HSS8	0.9	—	−0.2	—		
HSS9	0.8	—	0.1	—		
HSS10	0.7	—	—	0.2		
HSS11	0.7	—	—	0.5		
HSS12	0.7	—	—	0.7		
CR	1.0	0.9	0.9	0.9		
ωH	0.9	0.3	0.0	0.3		
ωH/CR	1.0	0.3	0.0	0.3		

Abbreviations: F1, Personalized stigma; F2, Public‐disclosure concerns; F3, Negative self‐image; HSS1‐HSS12, 12 items of the HPV stigma scale; CR, composite reliability; ωH, homogeneity coefficient; ECV, explained common variance; PUC, percentage of uncontaminated correlations.

### 3.5 GRM Analysis

**FIGURE 3:**
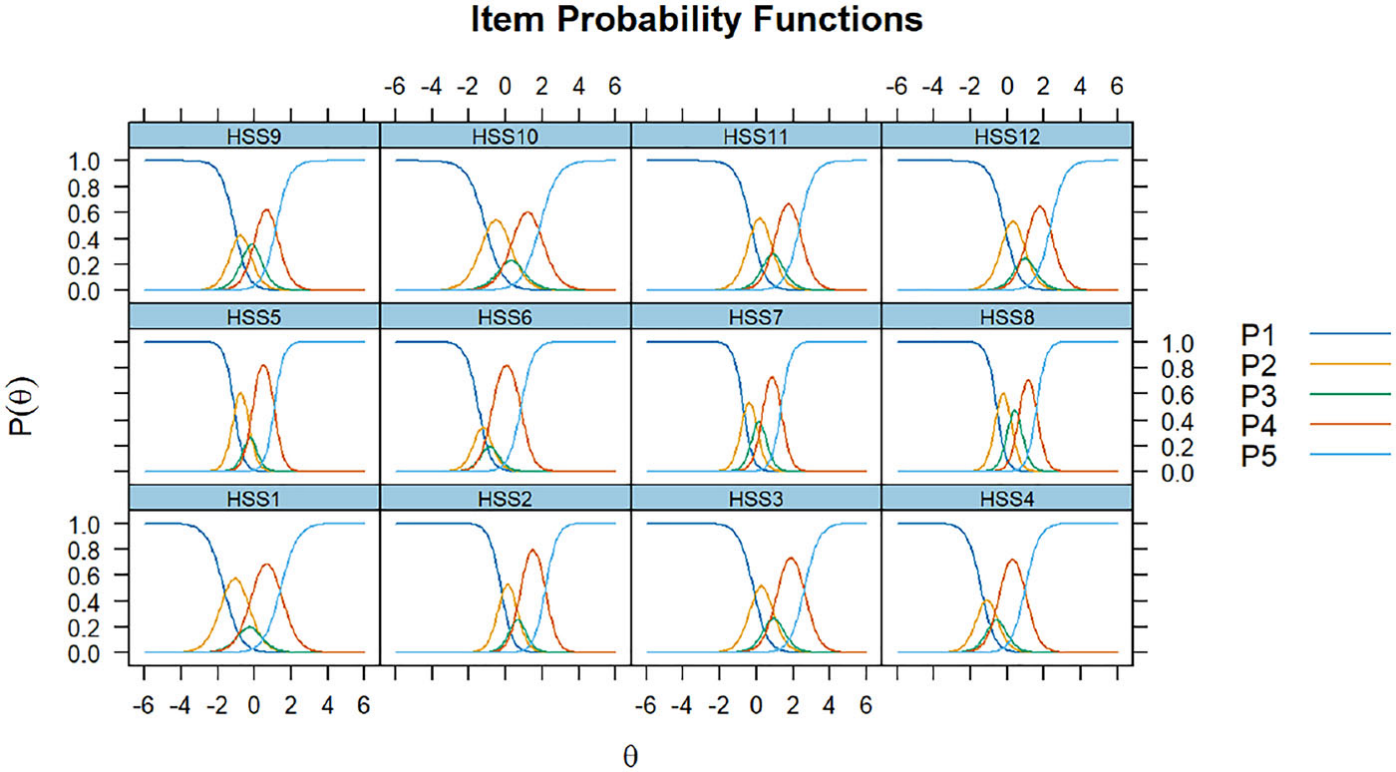
ICC. *Note*: HSS1‐HSS12: 12 items of the HPV stigma scale; P1–P5: 5 responses of each item.

**FIGURE 4:**
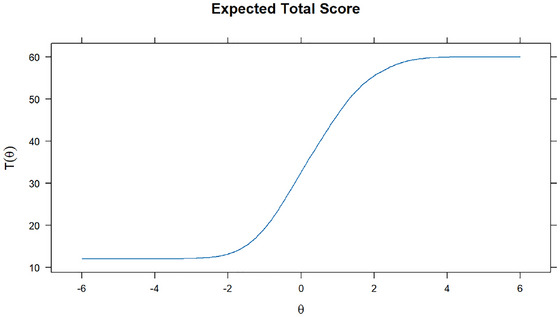
Expected total score plot.

**FIGURE 5:**
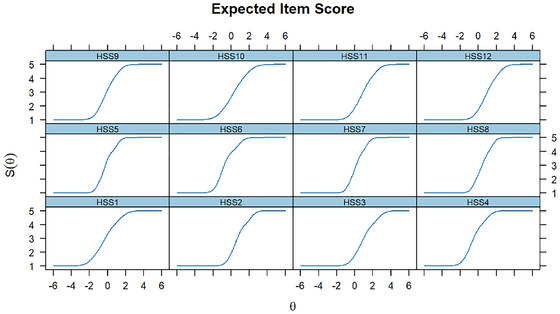
Expected item score plot. Note: HSS1‐HSS12: 12 items of the HPV stigma scale.

GRM was used to assess the relationship between participants’ stigma levels and scale total scores and item scores. Figure 3 presents the response probabilities of selecting each response category (1 to 5) for each item (HSS1 to HSS12) across different stigma levels (θ). The x‐axis represents the stigma level (θ), while the y‐axis represents the probability of selecting a particular response category (*P*(θ)). The ICC showed that those with higher levels of HPV stigma had a higher probability of selecting “5” for each item. Additionally, the expected total score and item score in Figures 4, 5 implied that individuals with higher levels of HPV stigma are expected to have higher total and item scores.

### 3.6 Latent Profile Analysis

**FIGURE 6:**
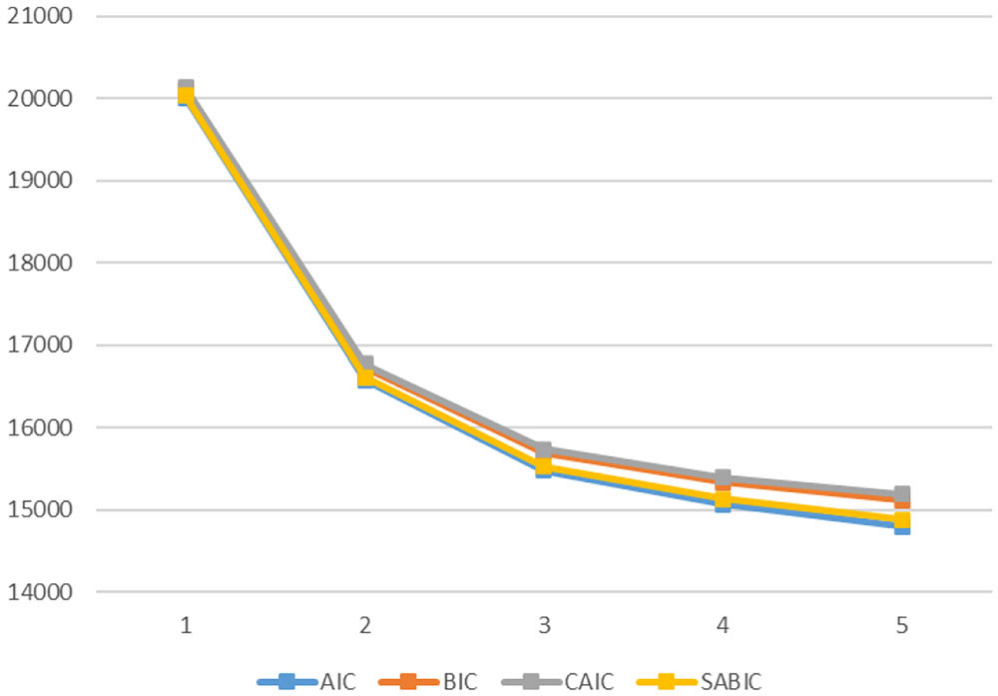
Elbow plot of the model fits in the five models.

**FIGURE 7:**
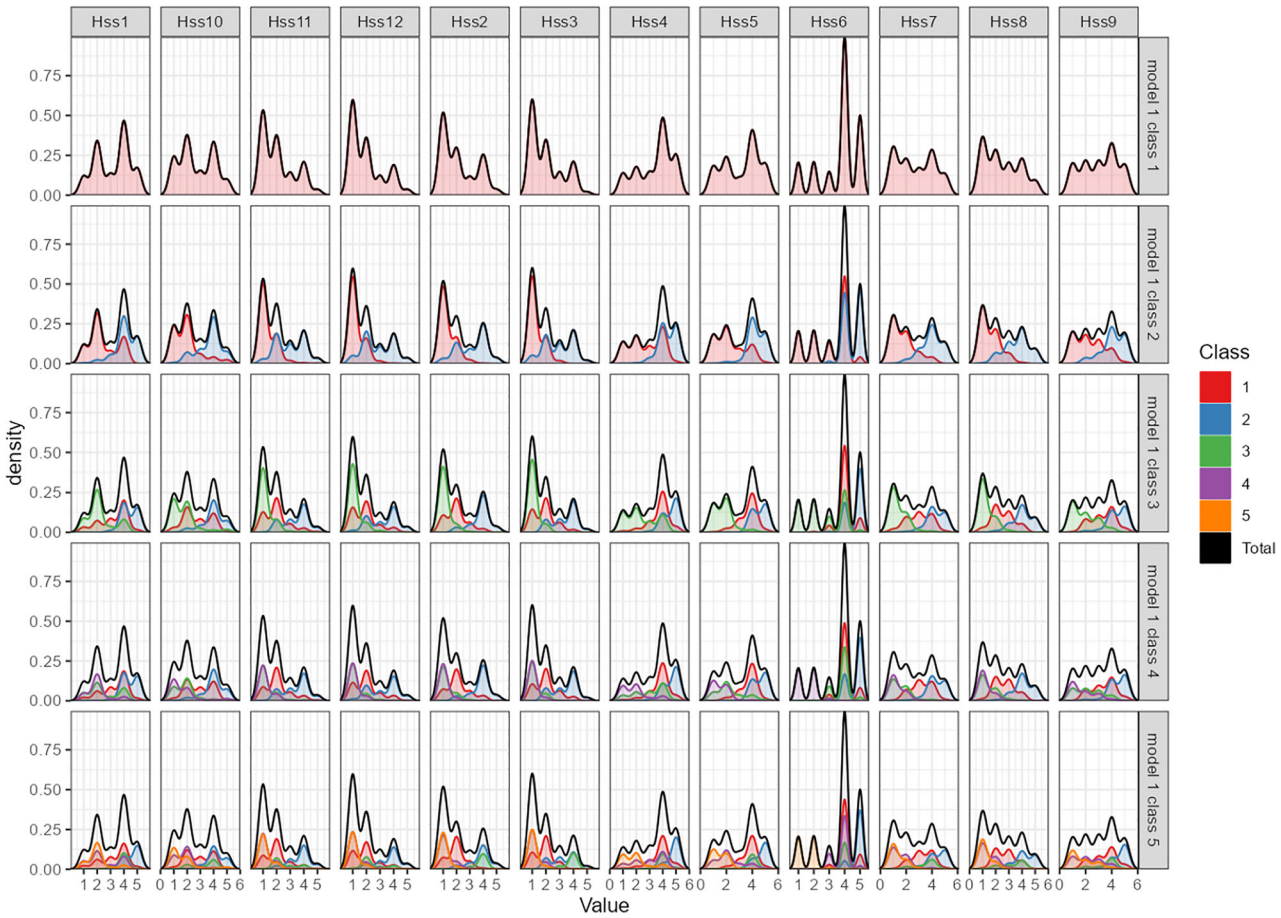
Density plot of the latent profiles with one to five classes. Note: HSS1‐HSS12: 12 items of the HPV stigma scale.

LPA is a person‐centered statistical method used to identify unobserved (latent) subgroups within a sample based on patterns of responses to multiple observed variables. LPA with 1–5 classes was conducted to further explore the characteristics of HPV stigma in women infected with HPV. The entropy values of the 5 models were all above 0.9, indicating high classification precision for each model. The AIC, BIC, CAIC, and SABIC values all showed a decreasing trend as the number of classes increased. However, the transition from one class to two classes resulted in the most significant decrease in the values of the information criteria, see Table 8 and Figure 6. Considering the parsimony and interpretability of the latent profiles, we finally determined the 2‐class model as optimal. The density of the latent profiles with 1 to 5 classes is illustrated in Figure 7.

**TABLE 8:** Comparison of the models of different classes.

Class	AIC	BIC	CAIC	SABIC	Entropy
1	20005	20106	20130	20030	1
2	16575	16731	16768	16614	0.957
3	15475	15686	15736	15527	0.955
4	15064	15329	15392	15129	0.952
5	14796	15117	15193	14876	0.948

**FIGURE 8:**
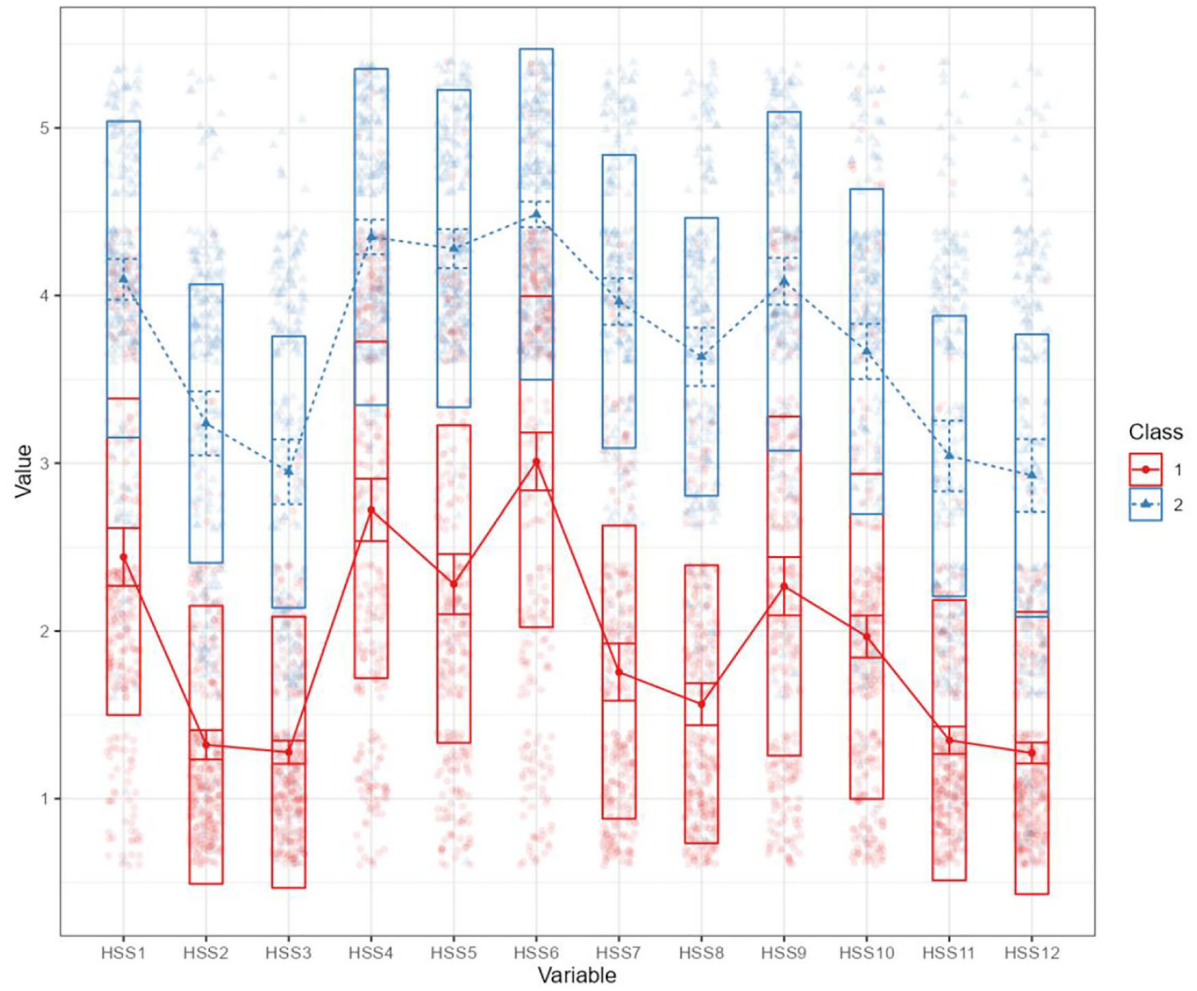
Latent profile plot of the 2‐class model. Note: HSS1‐HSS12: 12 items of the HPV stigma scale.

The characteristics of the two classes are presented in Figure 8. Given that the conditional means of all 12 items in Class 1 were lower than those in Class 2, and HPV stigma is a continuous psychological construct rather than a dichotomous “present/absent” trait. Class 1(sample size: 276) was labeled as the “low HPV stigma” group, and Class 2 (sample size: 225) was labeled as the “high HPV stigma” group. The optimal cut‐off value was determined to be 35 based on ROC analysis. A total score of 35 or above may indicate high levels of HPV stigma, with an area under the curve (AUC) of 0.999, a sensitivity of 99.10%, a specificity of 98.56%, and a Youden's index of 0.977, see Table 9. The Kappa coefficients between the LPA‐derived HPV stigma cutoff (total score ≥ 35) and the clinical cutoffs for moderate‐to‐severe depression (PHQ‐9 ≥ 10) and anxiety (GAD‐7 ≥ 10) were 0.429 and 0.415, respectively, indicating moderate agreement between high HPV stigma classification and the presence of clinically relevant emotional distress. To address the potential circularity from deriving the cutoff in the same sample used for LPA, we further implemented leave‐one‐out cross‐validation (LOOCV) to validate the cutoff's performance. The cross‐validated metrics included an AUC of 0.977, sensitivity of 0.986, specificity of 0.991, accuracy of 0.988, and a Kappa coefficient of 0.976, indicating the robustness of the cut‐off value of 35.

**TABLE 9:** Cut‐off values of the scale.

Cut‐off value	Sensitivity (%)	Specificity (%)	PPV (%)	NPV (%)	Youden's index	AUC
33	100%	93.17%	92.15%	100%	0.932	0.999
34	100%	95.68%	94.89%	100%	0.957	
35	99.10%	98.56%	98.22%	99.28%	0.977	
36	95.52%	100%	100%	96.53%	0.955	

Abbreviations: PPV, positive predictive value; NPV, negative predictive value; AUC, area under the curve.

To enhance clinical applicability, we also summarized the performance of the cutoff for each disease stage in Supplementary Table 11.

## 4 Discussion

Understanding the HPV stigma level in women infected with HPV is crucial for cervical cancer prevention, diagnosis, control, treatment, and rehabilitation since it influences patients’ willingness to get tested, seek treatment, disclose information, and adhere to treatment plans (Bae and Temkin 2025). However, currently we have no specific instrument to evaluate the stigma level among Chinese women infected with HPV. In this case, this study adapted and validated the 12‐item HPV‐SS designed for measuring stigma among women infected with HPV in China and provided evidence for its applicability in this population through a robust and rigorous methodological process.

In the initial validation through EFA, we extracted only 1 factor from the 12 items, indicating that the items of the HPV‐SS may reflect the overarching concept of HPV‐related stigma. Conceptually, the nature of HPV stigma could potentially be perceived as a unitary construct by the participants. A cross‐sectional study in Hong Kong, China has also supported the 1‐factor structure of stigmatizing beliefs towards women with high‐risk HPV infection (Kwan et al. 2012). However, the extraction of a single factor in EFA may not exclude the existence of a more nuanced factorial structure, since EFA is inherently data‐driven and may not capture the theoretical dimensionality of a construct. Hence, the subsequent CFA compared 1‐factor, 4‐factor, and 3‐factor models to rigorously test the theoretical structure of the HPV‐SS. The 4‐factor model was based on the original structure of the HIV‐SS from which the HPV‐SS was adapted, which allowed us to test whether the theoretically derived dimensions of personalized stigma, disclosure concerns, concerns about public attitudes, and negative self‐image could be replicated in the context of HPV stigma (Reinius et al. 2017). The 3‐factor model, on the other hand, was proposed based on the potential overlap between disclosure concerns and concerns about public attitudes by merging these two dimensions into “public‐disclosure concerns” to create a more parsimonious and conceptually coherent model. The decision to adopt the 3‐factor structure as optimal was substantiated through a comprehensive evaluation of reliability and validity metrics. Notably, the 4‐factor model also revealed strong associations between disclosure concerns and concerns about public attitudes, further supporting the rationality of consolidating them into a single dimension within the 3‐factor framework. Additionally, the items in both dimensions of disclosure concerns and concerns about public attitudes in the 4‐factor solution are intricately associated with the attitude to HPV stigma. Research has revealed that the risk associated with disclosing HPV status may be a result of negative public attitudes toward women infected with HPV (Bennett et al. 2020, 2024). The interconnection between them suggested that they are not distinct enough to be considered separate dimensions, thereby justifying their integration into the broader construct of public‐disclosure concerns. To ensure the robustness and generalizability of the factorial solution, factorial invariance was assessed across key demographic and clinical subgroups. The results supported full invariance, implying that the psychometric properties of the HPV‐SS were equivalent across groups, which supported the structural and measurement stability and cross‐group validity of the 3‐factor model.

Although FA confirmed the factorial structure of HPV‐SS, the practical application of this scale remains uncertain, particularly regarding whether evaluations should be based on the total score, dimension scores, or both. Therefore, a bi‐factor model incorporating both a general factor and specific factors was proposed to explore the optimal scoring method. The results supported the unidimensionality of the HPV‐SS, highlighting the presence of a general factor that integrates the dimensions and demonstrating that, at a broader level, the scale measures a unified construct of HPV stigma. Furthermore, the findings indicated that the total score is more meaningful than dimension scores when evaluating HPV stigma among women with HPV. In practice, using the total score may simplify the assessment process, enabling healthcare providers and researchers to quickly and comprehensively understand the overall level of stigma faced by women with HPV, particularly in resource‐limited settings or situations requiring efficiency (Rajkhowa et al. 2025). While the three specific factors complement the utility of total score by providing valuable insights into the distinct components contributing to the overall experience of HPV stigma, which may guide the development of targeted interventions on personalized stigma, public‐disclosure concerns, and negative self‐image, respectively, to effectively address the multifaceted nature of HPV stigma among women (Zheng et al. 2024).

In the IRT analyses, we conclude that a higher total score of the HPV‐SS represents higher levels of HPV stigma among women with HPV, consistent with the item settings. Further LPA and ROC analyses finally identified a cut‐off value of 35, where a total score of 35 or above indicates high levels of HPV stigma. The cut‐off value can be used as a screening tool to quickly identify women who may be experiencing high levels of HPV stigma in clinical settings, thereby facilitating timely intervention and support for their elevated risk of emotional distress and poor adherence to HPV care.

Despite the contributions of this study in validating the HPV‐SS among women infected with HPV in China, several limitations should be acknowledged. First, the sample was only recruited from Shenzhen, Guangdong Province, which may limit the external validity and generalizability of the findings due to China's regional variations in cultural norms related to stigma. However, Shenzhen's cultural diversity may still allow for a partial reflection of national trends. Second, although the HPV‐SS was developed through a rigorous validation process adapted from the HIV‐SS, it may not fully capture all dimensions or aspects of HPV‐related stigma, potentially overlooking nuanced experiences. Third, the cut‐off value for high HPV stigma was determined based on a sample from a maternity and child healthcare hospital, which may not fully represent the broader population, and a potential concern of circularity may be introduced, as we cannot fully exclude the possibility of logical overlap between the scale's item response patterns and its derived total scores. Given that public awareness of HPV and societal attitudes toward it are continually evolving, this cut‐off value may require periodic reassessment to remain relevant over time. Fourth, while most statistical indicators demonstrated strong performance, such as high internal consistency and AUC, their collective pattern of near‐optimal values warrants attention, since this could alternatively reflect either the scale's exceptionally strong psychometric properties or the presence of potential methodological issues such as overfitting that may have inflated these metrics. It should also be noted that, in certain analyses, some RMSEA values exceeded the ideal threshold. Nevertheless, these values remained near the margin of acceptability, and other fit indices consistently met the criteria for good model fit, supporting the overall validity of the factor structures within the context of this study. To further strengthen the evidence base, future research should prioritize large‐sample, multi‐center studies to enhance the scale's representativeness and evaluate its generalizability across diverse populations and settings. Independent external validation in distinct cohorts is also recommended. Additionally, longitudinal studies are needed to monitor temporal changes in HPV stigma levels and recalibrate the cut‐off value as necessary to reflect evolving societal perceptions.

## 5 Conclusion

This study validated the 12‐item HPV‐SS to assess the level of stigma experienced by women infected with HPV in China, thereby establishing its applicability, reliability, and validity within this population. Relevant departments and healthcare professionals can utilize this instrument to evaluate the stigma levels faced by women with HPV and take targeted interventions for those with high levels of stigma to improve cervical cancer prevention and control efforts while enhancing the psychological well‐being of affected women. Further research is also needed to evaluate the practical utility of this HPV stigma scale in clinical practice settings.

## Author Contributions

ZD and SJ prepared the first draft. XS, ZL, and YQ provided overall guidance, and managed the overall project. ZD, SJ, HH, TY, YW, DC, FG, and XH were responsible for the questionnaire survey and data analysis.

## Funding

This work was supported by CAMS Innovation Fund for Medical Sciences (CIFMS) (2021‐I2M‐1‐004) and Sanming Project of Medicine in Shenzhen (SZSM202211032).

## Ethics Statement

This study has been approved by the Ethics Committee of the Chinese Academy of Medical Science (ID: CAMS&PUMC‐IEC‐2024‐001) on January 12th, 2024.

## Conflicts of Interest

The authors declare no conflict of interest.

## Supporting information



Supplementary Tables: brb371044‐sup‐0001‐TableS1‐S11.docx

## Data Availability

The data that support the findings of this study are available from the corresponding author, upon reasonable request.
