# Supplementary material for: Validation of the Chinese Version of the HPV Stigma Scale: A Multidimensional Analysis in Women With HPV
Source: Brain Behav. 2025 Nov 11;15(11):e71044. doi: 10.1002/brb3.71044 (PMC12605962; doi:10.1002/brb3.71044)
Supplement: Supplementary file 1 — Supplementary Tables: brb371044‐sup‐0001‐TableS1‐S11.docx [file BRB3-15-e71044-s001.docx]

**Supplementary Table 1.** Item details and factor loadings of the exploratory factor analysis

| **Item** | **Label** | **Factor loading** |
| --- | --- | --- |
| Some people avoid touching me once they know I have HPV | HSS1 | 0.8 |
| People I care about stopped calling after learning I have HPV | HSS2 | 0.8 |
| I have lost friends by telling them I have HPV | HSS3 | 0.8 |
| Telling someone I have HPV is risky | HSS4 | 0.8 |
| I work hard to keep my HPV a secret | HSS5 | 0.9 |
| I am very careful who I tell that I have HPV | HSS6 | 0.8 |
| People with HPV are treated like outcasts | HSS7 | 0.9 |
| Most people believe a person who has HPV is dirty | HSS8 | 0.9 |
| Most people are uncomfortable around someone with HPV | HSS9 | 0.8 |
| I feel guilty because I have HPV | HSS10 | 0.7 |
| People’s attitudes about HPV make me feel worse about myself | HSS11 | 0.8 |
| I feel I’m not as good a person as others because I have HPV | HSS12 | 0.8 |

**Supplementary Table 2.** Parameter estimates, reliability, and convergent validity of the 1-factor model

| **Indicator** | **Estimate** | **SE** | **β** | **z** | **p** | **CR** | **AVE** |
| --- | --- | --- | --- | --- | --- | --- | --- |
| HSS1 | 1.0 |  | 0.7 |  |  | 0.9 | 0.6 |
| HSS2 | 1.4 | 0.1 | 0.8 | 12.2 | <0.001 |  |  |
| HSS3 | 1.2 | 0.1 | 0.8 | 11.7 | <0.001 |  |  |
| HSS4 | 1.0 | 0.1 | 0.7 | 9.9 | <0.001 |  |  |
| HSS5 | 1.4 | 0.1 | 0.8 | 12.1 | <0.001 |  |  |
| HSS6 | 1.1 | 0.1 | 0.7 | 10.7 | <0.001 |  |  |
| HSS7 | 1.5 | 0.1 | 0.9 | 12.5 | <0.001 |  |  |
| HSS8 | 1.4 | 0.1 | 0.9 | 12.5 | <0.001 |  |  |
| HSS9 | 1.2 | 0.1 | 0.7 | 10.9 | <0.001 |  |  |
| HSS10 | 1.1 | 0.1 | 0.7 | 10.2 | <0.001 |  |  |
| HSS11 | 1.1 | 0.1 | 0.8 | 11.3 | <0.001 |  |  |
| HSS12 | 1.0 | 0.1 | 0.7 | 10.9 | <0.001 |  |  |

Note: HSS1-HSS12: 12 items of the HPV stigma scale; CR: composite reliability; AVE: average variance extracted

**Supplementary Table 3.** Parameter estimates, reliability, and convergent validity of the 4-factor model

| **Factor** | **Indicator** | **Estimate** | **SE** | **β** | **z** | **p** | **CR** | **AVE** |
| --- | --- | --- | --- | --- | --- | --- | --- | --- |
| F1 | HSS1 | 1.0 |  | 0.6 |  |  | 0.9 | 0.8 |
|  | HSS2 | 1.7 | 0.1 | 1.0 | 12.4 | <0.001 |  |  |
|  | HSS3 | 1.5 | 0.1 | 0.9 | 12.1 | <0.001 |  |  |
| F2 | HSS4 | 1.0 |  | 0.7 |  |  | 0.8 | 0.6 |
|  | HSS5 | 1.4 | 0.1 | 0.9 | 13.1 | <0.001 |  |  |
|  | HSS6 | 1.1 | 0.1 | 0.8 | 11.8 | <0.001 |  |  |
| F3 | HSS7 | 1.0 |  | 0.9 |  |  | 0.9 | 0.7 |
|  | HSS8 | 0.9 | 0.0 | 0.9 | 22.5 | <0.001 |  |  |
|  | HSS9 | 0.8 | 0.1 | 0.8 | 15.7 | <0.001 |  |  |
| F4 | HSS10 | 1.0 |  | 0.7 |  |  | 0.9 | 0.7 |
|  | HSS11 | 1.3 | 0.1 | 0.9 | 12.5 | <0.001 |  |  |
|  | HSS12 | 1.2 | 0.1 | 0.9 | 12.3 | <0.001 |  |  |

Note: F1: Personalized stigma; F2: Disclosure concerns; F3: Concerns about public attitudes; F4: Negative self-image; HSS1-HSS12: 12 items of the HPV stigma scale; CR: composite reliability; AVE: average variance extracted

**Supplementary Table 4.** Heterotrait-monotrait ratio of correlations in the 4-factor model

|  | **F1** | **F2** | **F3** | **F4** |
| --- | --- | --- | --- | --- |
| F1 | 1 |  |  |  |
| F2 | 0.855 | 1 |  |  |
| F3 | 0.866 | 0.909 | 1 |  |
| F4 | 0.821 | 0.797 | 0.830 | 1 |

Note: F1: Personalized stigma; F2: Disclosure concerns; F3: Concerns about public attitudes; F4: Negative self-image

**Supplementary Table 5.** Multiple-Indicator Multiple-Cause model on the measurement invariance of HPV Stigma Scale across demographic characteristics

| **Variables** | **β** | **S.E.** | **Z** | **P** |
| --- | --- | --- | --- | --- |
| **Age** | 0.0 | 0.0 | 0.5 | 0.603 |
| **Ethnicity** |  |  |  |  |
| Han | 0.0 |  |  |  |
| Other | 0.1 | 0.1 | 1.2 | 0.226 |
| **Permanent residence in the past 6 months** |  |  |  |  |
| Urban area | 0.0 |  |  |  |
| Rural area | 0.1 | 0.2 | 0.5 | 0.588 |
| **Religion** |  |  |  |  |
| No | 0.0 |  |  |  |
| Yes | 0.0 | 0.1 | 0.4 | 0.708 |
| **Degree** |  |  |  |  |
| Junior high school degree or below | 0.0 |  |  |  |
| Senior high school or secondary vocational school degree | -0.1 | 0.1 | -0.7 | 0.496 |
| Associate degree | -0.3 | 0.1 | -2.1 | 0.038 |
| Bachelor’s degree | -0.6 | 0.2 | -3.6 | <0.001 |
| Master's or doctor’s degree | -0.8 | 0.2 | -3.9 | <0.001 |
| **Marriage Status** |  |  |  |  |
| Single/unmarried | 0.0 |  |  |  |
| Married | -0.2 | 0.1 | -1.6 | 0.099 |
| Divorced | 0.2 | 0.2 | 1.1 | 0.292 |
| **Annual household income (ten thousand yuan)** |  |  |  |  |
| ≤5 | 0.0 |  |  |  |
| 5-10 | 0.2 | 0.2 | 0.7 | 0.482 |
| 10-30 | 0.1 | 0.2 | 0.4 | 0.701 |
| >30 | 0.0 | 0.2 | 0.2 | 0.864 |
| **Alcohol** |  |  |  |  |
| No | 0.0 |  |  |  |
| Yes | -0.2 | 0.1 | -3.4 | 0.001 |
| **Number of children** |  |  |  |  |
| 0 | 0.0 |  |  |  |
| 1 | 0.0 | 0.1 | -0.3 | 0.779 |
| 2 | 0.0 | 0.1 | -0.3 | 0.791 |
| ≥3 | 0.1 | 0.2 | 0.7 | 0.473 |
| **Number of sexual partners** |  |  |  |  |
| 0 | 0.0 |  |  |  |
| 1 | 0.2 | 0.3 | 0.7 | 0.479 |
| ≥2 | 0.4 | 0.3 | 1.5 | 0.137 |
| **HPV vaccination** |  |  |  |  |
| No |  |  |  |  |
| Yes | -0.2 | 0.1 | -2.6 | 0.009 |
| **Regular cervical cancer screening before diagnosis** |  |  |  |  |
| No | 0.0 |  |  |  |
| Yes | -0.4 | 0.1 | -4.2 | <0.001 |
| **Discomfort during the treatment** |  |  |  |  |
| No | 0.0 |  |  |  |
| Yes | 0.3 | 0.1 | 3.8 | <0.001 |

Note: S.E.: Standard error

**Supplementary Table 6.** Multi-group analyses on degree

| Model | Δχ^2^ | Δdf | P | ΔTLI |
| --- | --- | --- | --- | --- |
| Baseline |  |  |  |  |
| Metric invariance | 57.4 | 36 | 0.013 | -0.013 |
| Scalar invariance | 89.6 | 48 | <0.001 | -0.01 |
| Measurement residual variance invariance | 234.5 | 48. | <0.001 | 0.018 |
| Factor variance and covariance invariance | 52.0 | 24 | <0.001 | -0.003 |
| Latent mean invariance | 182.0 | 12 | <0.001 | 0.024 |

Note: df: degree of freedom; TLI: Tucker-Lewis Index

**Supplementary Table 7.** Multi-group analyses on alcohol use

| Model | Δχ^2^ | Δdf | P | ΔTLI |
| --- | --- | --- | --- | --- |
| Baseline |  |  |  |  |
| Metric invariance | 11.3 | 9 | 0.255 | -0.008 |
| Scalar invariance | 9.2 | 12 | 0.687 | -0.011 |
| Measurement residual variance invariance | 46.0 | 12 | <0.001 | -0.002 |
| Factor variance and covariance invariance | 33.9 | 6 | <0.001 | 0.002 |
| Latent mean invariance | 32.9 | 3 | <0.001 | 0.003 |

Note: df: degree of freedom; TLI: Tucker-Lewis Index

**Supplementary Table 8.** Multi-group analyses on HPV vaccination

| Model | Δχ^2^ | Δdf | P | ΔTLI |
| --- | --- | --- | --- | --- |
| Baseline |  |  |  |  |
| Metric invariance | 35.8 | 9 | <0.001 | -0.003 |
| Scalar invariance | 17.5 | 12 | 0.132 | -0.011 |
| Measurement residual variance invariance | 171.3 | 12 | <0.001 | 0.021 |
| Factor variance and covariance invariance | 4.9 | 6 | 0.556 | -0.004 |
| Latent mean invariance | 121.0 | 3 | <0.001 | 0.02 |

Note: df: degree of freedom; TLI: Tucker-Lewis Index

**Supplementary Table 9.** Multi-group analyses on cervical cancer screening before diagnosis

| Model | Δχ^2^ | Δdf | P | ΔTLI |
| --- | --- | --- | --- | --- |
| Baseline |  |  |  |  |
| Metric invariance | 42.2 | 9 | <0.001 | -0.002 |
| Scalar invariance | 17.6 | 12 | 0.128 | -0.011 |
| Measurement residual variance invariance | 85.0 | 12 | <0.001 | 0.004 |
| Factor variance and covariance invariance | 10.9 | 6 | 0.091 | -0.004 |
| Latent mean invariance | 72.3 | 3 | <0.001 | 0.011 |

Note: df: degree of freedom; TLI: Tucker-Lewis Index

**Supplementary Table 10.** Multi-group analyses on discomfort during treatment.

| Model | Δχ^2^ | Δdf | P | ΔTLI |
| --- | --- | --- | --- | --- |
| Baseline |  |  |  |  |
| Metric invariance | 18.1 | 9 | 0.034 | -0.007 |
| Scalar invariance | 23.9 | 12 | 0.021 | -0.008 |
| Measurement residual variance invariance | 71.7 | 12 | <0.001 | 0.003 |
| Factor variance and covariance invariance | 18.0 | 6 | 0.006 | -0.002 |
| Latent mean invariance | 33.5 | 3 | <0.001 | 0.003 |

Note: df: degree of freedom; TLI: Tucker-Lewis Index

**Supplementary Table 11.** Cutoff performance for each disease stage

| **Disease stage** | **Sensitivity (%)** | **Specificity (%)** | **PPV (%)** | **NPV (%)** | **Youden's index** | **Accuracy** |
| --- | --- | --- | --- | --- | --- | --- |
| HPV infection | 100% | 99.12% | 94.74% | 100% | 0.991 | 0.992 |
| Low-grade squamous intraepithelial lesions | 100% | 97.80% | 97.44% | 100% | 0.978 | 0.988 |
| High-grade squamous intraepithelial lesions | 98.13% | 98.53% | 99.06% | 97.10% | 0.967 | 0.983 |
| Cervical cancer | 100% | 100% | 100% | 100% | 1.000 | 1.000 |

Note: PPV: positive predictive value; NPV: negative predictive value; AUC: area under the curve
